# Virtual fracture clinics: be vigilant for the ligamentous elbow injury

**DOI:** 10.1093/jscr/rjac587

**Published:** 2022-12-17

**Authors:** Eve E Robertson-Waters, Rory Cuthbert, Lee Van Rensburg

**Affiliations:** Department of Trauma and Orthopaedic surgery, Addenbrooke’s Hospital, Cambridge CB2 0QQ, UK; Department of Surgery, Division of Trauma and Orthopaedic Surgery, University of Cambridge, Addenbrooke’s Hospital, Cambridge CB2 0QQ, UK; Department of Trauma and Orthopaedic surgery, Addenbrooke’s Hospital, Cambridge CB2 0QQ, UK; Department of Trauma and Orthopaedic surgery, Addenbrooke’s Hospital, Cambridge CB2 0QQ, UK

## Abstract

The Covid-19 pandemic encouraged remote healthcare and led to dependency on virtual fracture clinics (VFC). VFC are orthopaedic consultant-led clinics where cases are reviewed virtually following referral by emergency department clinicians. Success is contingent on a comprehensive initial history and examination. This pathway has high patient satisfaction rates and cost-saving benefits. However, clinicians must be vigilant for high-energy mechanisms or examination findings suggestive of greater underlying injury. In this case, VFC missed a rare ipsilateral annular ligament injury in a 15-year old with an undisplaced radial neck fracture, following a fall from a horse. This led to radial head dislocation and delayed surgical repair. Untreated, radial head dislocations lead to pain and reduced range of movement. Despite the rarity of this injury pattern, face-to-face orthopaedic examination would have raised concern for significant ligamentous injury. A high-energy mechanism of injury mandates face-to-face senior orthopaedic review to avoid missing serious concomitant injury.

## INTRODUCTION

The need for remote healthcare led to increased dependency on virtual fracture clinics (VFC) during the COVID-19 pandemic. VFCs continue to be used nationwide and have proven to be an effective tool for streamlining trauma services and improving compliance with British Orthopaedic Association (BOA) guidelines, which endorse review in fracture clinic within 72 h of acute traumatic injury [1].

VFC is an orthopaedic consultant-led clinic where case histories and radiographs are reviewed virtually following referral by emergency department clinicians. Patients are subsequently booked into face-to-face fracture clinics or discharged with advice. This is particularly advised for a number of common fracture patterns, including minimally displaced radial head/neck fractures. The success of VFC is contingent on a comprehensive history and examination in the emergency department. However, clinicians must be vigilant for high-energy mechanisms or examination findings suggestive of greater underlying injury.

We present the case and accompanying video of a 15-year-old female who sustained an annular ligament injury, which was missed following the VFC referral. We conclude that a history of a high-energy mechanism of injury or clinical signs suggestive of significant ligamentous injury mandates a face-to-face follow-up.

## CASE REPORT

**Figure 1:**
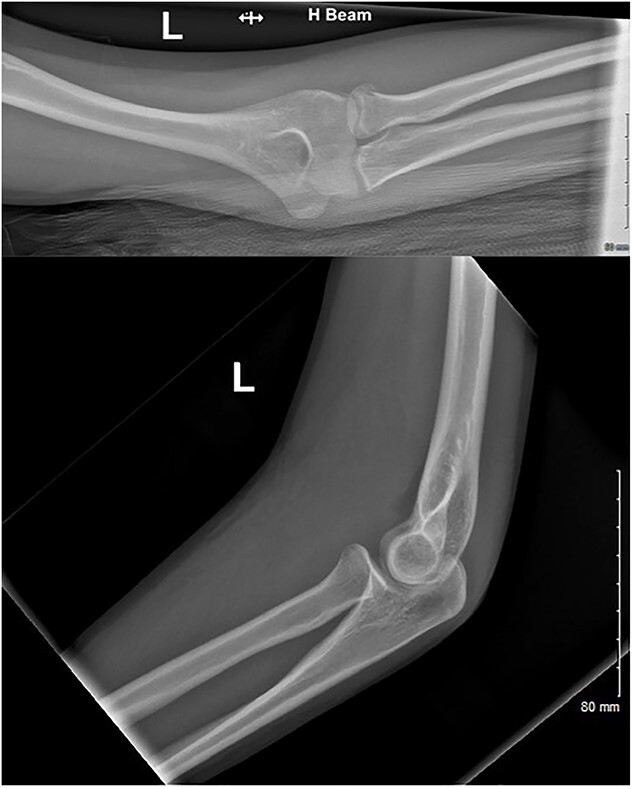
AP and lateral radiographs of the injury at first presentation to the emergency department.

A 15-year-old right-hand-dominant female fell from her horse onto an outstretched left hand (elbow extended, forearm pronated) at an equestrian event (Video 1). The patient immediately attended the emergency department with pain and significant swelling over the medial and lateral aspect of the elbow. Radiographs were performed (Fig. 1) and an undisplaced radial neck fracture was identified. As per guidance for non-displaced radial neck fractures, the patient was discharged with a broad-arm sling and a follow-up appointment was scheduled in VFC. At VFC, the patient was discharged with a plan to mobilize as the pain allows, avoid lifting for 2 months and to return if experiencing limitation in the range of movement.

**Figure 2:**
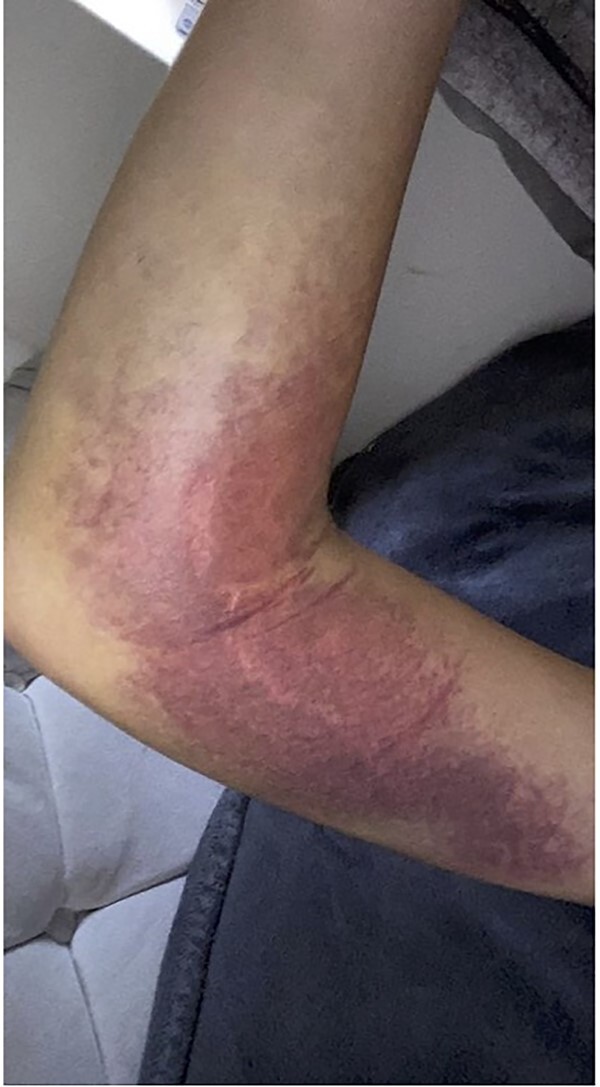
Haematoma evident at day-1 post-injury.

**Figure 3:**
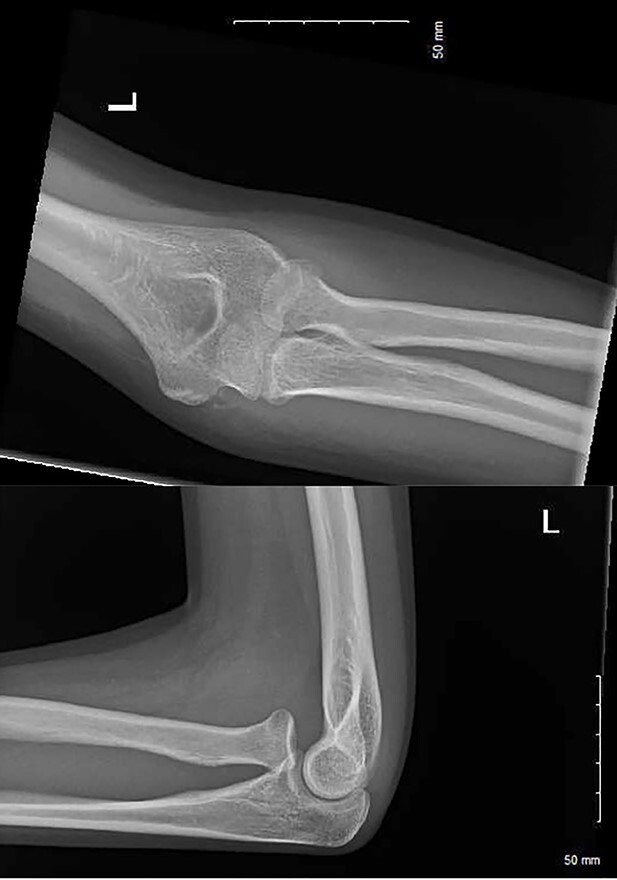
AP and lateral radiographs at 2 months post-injury.

After 1 month, the patient arranged a face-to-face follow-up due to ongoing pain, extensive haematoma and limited range of movement (Fig. 2). On assessment by an orthopaedic consultant, the range of movement was limited to 30–100°, supination to 50° and full pronation. Repeat radiographs demonstrated the radial head had now dislocated anteriorly, suggestive of annular ligament rupture (Fig. 3).

**Figure 4:**
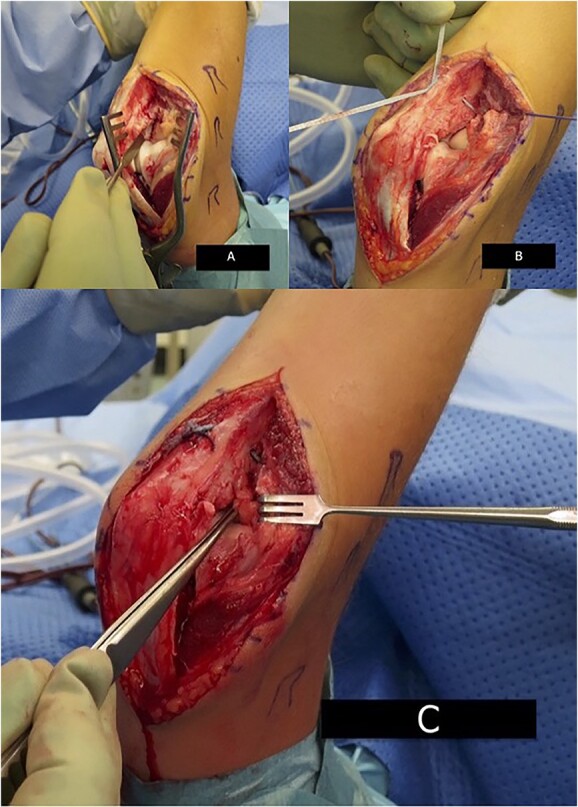
Intra-operative image demonstrating annular ligament reconstruction. (**A**) Surgical view via Boyd approach demonstrating ruptured annular ligament and LUCL. (**B**) No.2 Fibertape suture reconstruction. (**C**) Forceps demonstrate reconstructed LUCL and annular ligament.

At 6 weeks post-injury, surgery was performed via a Boyd approach. The annular ligament and lateral ulnar collateral ligament (LUCL) were ruptured. The radial neck fracture had healed. An internal brace construct using No.2 Fibertape suture was developed around the radial head and neck and secured via drill-holes at the origin and insertion of the annular ligament (Fig. 4). The LUCL and annular ligament tissue quality remained reasonable and both were reattached to the ulna via transosseous drill-holes. Post-repair, the elbow was stable throughout the arc of movement. The full range of movement was encouraged post-operatively with no load-bearing exercises for 6 weeks.

At 8 months post-operatively, the patient has resumed full activities (including horse-riding) with no ongoing pain or limitation in the range of movement.

## DISCUSSION

In the UK, cases initially assessed by emergency department practitioners are commonly referred to VFC, where radiographs are reviewed remotely within 72 h by a consultant-led team. At VFC, patients are either discharged with advice, referred for physiotherapy, or triaged to a subspecialty. VFC has streamlined trauma services since its inception in 2011, and is the BOA recommended pathway, with high patient satisfaction rates and cost-saving benefits [2] emphasized by the COVID-19 pandemic. In some trusts, common fracture patterns such as undisplaced radial head/neck fractures are deemed suitable for direct discharge from VFC with advice leaflets on expected recovery. The VFC pathway is mostly successful, with an infrequent need for unplanned face-to-face consultation and low reported late surgical intervention rates of 0.1–1% [3, 4]. However, we contend that in the case of high-energy trauma, even where there is minimal radiographic fracture displacement, there should be a low threshold for referral to the on-call orthopaedic team or facilitation of face-to-face fracture clinic review to avoid missing concomitant injury.

The incidence of radial head and neck fractures is 55 per 100 000 population. The mean age of injury is 40 years [5], commonly following a fall on an outstretched hand with an extended elbow and supinated forearm. The majority are stable, minimally displaced injuries. Displaced fractures are more likely to occur concomitantly with another injury to the elbow, including fractures of the proximal ulna [5], which may not be initially evident. As this case demonstrates, the lateral collateral ligament complex can also be concomitantly injured. This complex comprises four ligaments (annular ligament, lateral ulna collateral ligament, lateral radial collateral ligament and accessory lateral collateral ligament) that stabilize the elbow against varus and external rotation stress. Further, the annular ligament plays an additional role as the primary stabilizer of the radial head within the proximal ulna’s lesser sigmoid notch during forearm pronosupination. Therefore, annular ligament rupture can cause radial head dislocation. Untreated dislocation is associated with a reduced range of movement, power, dexterity and pain [6]. In the case of late presentation, surgical options include osteotomy and radial head excision/replacement alongside ligamentous reconstruction, with various techniques employed, from autologous grafting to synthetic tape with bone anchors. As this case demonstrates, excellent post-operative functional outcomes can be achieved.

The injury pattern in our case (ipsilateral radial neck fracture with annular ligament rupture) is rare—most traumatic radial head dislocations occur as part of a Monteggia fracture pattern. Despite its rarity, orthopaedic examination would have immediately raised concern for significant ligamentous injury given the extensive haematoma (Fig. 2) and limitation in the range of movement evident. VFC advice leaflets for all elbow injuries must underscore the need for face-to-face consultation if significant bruising or limitation in the range of movement persists beyond 1 week post-injury.

In conclusion, although VFC have proven an effective tool for streamlining trauma services, high-energy injury mechanism mandates need a face-to-face orthopaedic review to avoid missing concomitant injury. Further, VFC guidance for all elbow injuries must reiterate the requirement for face-to-face orthopaedic consultation if clinical symptoms suggestive of ligamentous injury (such as significant haematoma or limitation in the range of movement) persist beyond 1 week post-injury.

## Supplementary Material

Video_1_rjac587
